# Evaluation of efficacy of serological methods for detection of HCV infection in blood donors: A single centre experience

**DOI:** 10.12669/pjms.345.15707

**Published:** 2018

**Authors:** Arshi Naz, Samina Naz Mukry, Imran Naseer, Tahir Sultan Shamsi

**Affiliations:** 1 Arshi Naz, PhD. National Institute of Blood Diseases & Bone Marrow Transplantation, Karachi, Pakistan; 2 Samina Naz Mukry, PhD. National Institute of Blood Diseases & Bone Marrow Transplantation, Karachi, Pakistan; 3 Imran Naseer, B.Sc. National Institute of Blood Diseases & Bone Marrow Transplantation, Karachi, Pakistan; 4 Tahir Sultan Shamsi, FRC Path. National Institute of Blood Diseases & Bone Marrow Transplantation, Karachi, Pakistan

**Keywords:** Active HCV infection, Blood donors

## Abstract

**Background and Objective:**

Blood transfusion is an essential and life-saving medical intervention. Despite multiple preventive measures transfusion-transmitted hepatitis C virus (HCV) infection continues to be a major healthcare issue in Pakistan. This study was conducted at National Institute of Blood Diseases & Bone Marrow Transplantation to evaluate the frequency of active HCV infection with or without co-infection in blood donors and also to determine comparative efficacy of Multisure HCV antibody assay (MHAA); a new serological device.

**Methods:**

A total of 14652 blood donors visiting National Institute of Blood Diseases & Bone Marrow Transplantation (NIBD) Blood Bank from January 2013 to July 2014 were enrolled and screened for a range of blood borne infections such as HBV, HCV, HIV, malaria and syphilis. The HCV was screened simultaneously by Abbot Architect anti-HCV assay (CLIA) and MHAA. The active HCV infection was confirmed by nucleic acid testing (NAT) in reactive donors. Later; for determination of comparative efficacy of MHAA; all NAT positive samples were further tested using Monolisa™, HCV blot 3.0, Anti-HCV plus V2 and Anti-HCV-MPBIO-EIA.

**Results:**

The HCV reactive sera were observed in 1.563% (226) donors. The NAT confirmed active HCV infection in 138 donors. Overall 27.84% of HCV positive donors exhibited co-infection either with HBV (2.57%), syphilis (22.78%). Triple infection was not observed in any donor. The efficacy of MHAA is comparable to all the serological tests with a sensitivity of about 96.89%.

**Conclusion:**

Active HCV infection was present in 0.94% donors. With a sensitivity of 96.89% (95% CI: 95.66-98.12) the multi-parametric device MHAA can effectively detect HCV infection in donors. Thus, it can be used in limited health care settings for HCV screening.

## INTRODUCTION

Liver disease due to Hepatitis C virus (HCV) is a common global infection.^1^ Out of the estimated 130-170 million HCV affected people about 8.6 millions are Pakistanis.^2^,^3^ HCV is a positive sense single stranded RNA virus discovered in 1980’s as non-A and non-B hepatitis virus. Due to its unique characteristics a separate genus *Hepacivirus* has been defined for HCV. It shares most characters with genus *Flavivirus* and *Pestivirus* of family Flaviviridae.^4^ The HCV infection may either be acute, chronic or chronic carrier state disease. The clinical manifestation for acute hepatitis by HCV is characterized by mild or asymptomatic infection which might lead to chronic severe form of liver disease, cirrhosis and hepatocellular carcinoma. Considerable rate of mortality (0.5 million/ year) due to HCV cirrhosis or liver cancer is reported worldwide.^5^ An individual’s immune system plays an important role in clearing HCV infections. In about 15-30% cases acute/short term infection occur; while the remaining 70-85% cases may have chronic infection which may persist even after treatment leading to liver cancer in 15-20% cases worldwide. In the economically and technologically advanced countries most of the liver transplants are due to HCV infections.^6^,^7^ Even after liver transplant about 4% cases results in death. The mode of transmission of HCV is by the reuse of unsterilized syringes and/or medical equipment, transfusion of contaminated blood or blood products and contaminated instruments for ear and nose piercing, shaving etc. It may be transmitted via placenta to the developing fetus if the mother is infected.^8^

Various diagnostic tools have been developed for the diagnosis and management of HCV infection in general population. These tools can be broadly characterized as indirect and direct diagnostic tests. In case of indirect tests the HCV infection or exposure to HCV is indirectly detected; based on the presence or absence of antibodies (IgM or IgG) to HCV.^9^ One of the limitations of indirect assays is their inability to discriminate between active or past infection. With the technological advancement most of the serological assays have been automated and their sensitivity and specificity has also been improved by the use of many recombinant HCV antigens from the core, NS3, NS4 and NS5 regions.^10^ On the other hand direct assays are more accurate. The direct assays can detect HCV core antigen or HCV genome by molecular assays. The Abbott Architect chemiluminescence immune-assay (CLIA) can detect and quantify HCV core antigen in an automated manner.^11^ It can be effectively used in resource constrained settings. However, it is less sensitive than the HCV RNA detection by PCR which is the gold standard for HCV detection and monitoring of treatment as per guidelines by WHO.^12^ Over the time, several combination assays like Monolisa antigen/antibody Ultra have been developed which can simultaneously detect antibodies and core antigen of HCV. These combination assays have improved the HCV detection. The virus can now be easily detected during the window period of antibody assays.^13^

The pre-transfusion screening of donors with sensitive assays to detect active HCV infection is a preventive measure for the control of transfusion dependent spread of HCV. The WHO lays especial emphasis on screening of blood and blood products for all transfusion transmissible infections (TTI) such as HCV, HBV, HIV, syphilis, malaria etc.^12^ In most blood centers with limited laboratory setup rapid devices are generally used for initial screening of infected donors. These rapid tests are cheap alternative of combination or third generation enzyme immune-assays (EIA).^14^

This study was conducted to evaluate the frequency of active HCV infection with or without co-infection in blood donors. The comparative efficacy of a new serological device for HCV screening (Multisure HCV antibody assay) was also determined. Multisure HCV antibody assay is developed for the detection and differentiation of HCV antibodies into core, NS3, NS4 and NS5.

## METHODS

This cross sectional study was conducted at the Department of Immunology, National Institute of Blood Diseases & Bone Marrow Transplantation (NIBD) after approval by ethical review committee of NIBD Karachi. The study protocol adhered to the tenets of the Declaration of Helsinki.

### Blood Donors

A total of 14652 blood donors attending NIBD Blood Bank from January 2013 to July 2014 were included in this study. Both exchange and voluntary blood donors were enrolled. Following WHO standard guidelines of blood and blood product screening; all donors were screened for HBV, HCV, HIV, syphilis and malaria. Screening for HBV, HIV and syphilis was done by chemiluminescence assay using Abbot Architect while malaria and filariasis were confirmed by microscopy of Leishman’s stained blood films.

### HCV Screening of Donors

The HCV was screened simultaneously by Abbott Architect anti-HCV assay (CLIA) and Multisure HCV antibody assay (MHAA) in all the donors. The HCV reactive patients by any of these assays were further screened for active HCV infection by the gold standard HCV nucleic acid testing (NAT) using artus^®^ RG RT-PCR kit.

### Determination of comparative efficacy of MHAA

In order to study the efficacy of MHAA; about 250 samples including both positive and negative samples by NAT were further tested using standard serological assays including ELISA by Monolisa™ Anti-HCV Plus V2 and two different EIAs i.e. Anti-HCV-MPBIO-EIA and MPD HCV blot 3.0.

### Statistical analysis

The SPSS version 20 was used to analyze the data. The sensitivity, specificity, positive predictive value (PPV) and negative predictive values (NPV) were also calculated with PCR as gold standard. The Youden’s J index was also estimated for comparative performance analysis of different tests.

## RESULTS

**Fig.1:**
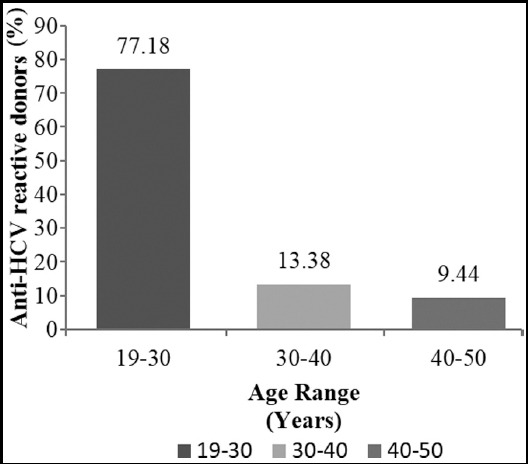
Age distribution of Anti-HCV reactive donors. All Anti-HCV reactive donors were categorized into three age groups; 19-30, 30-40 and 40-50 years respectively. The frequency in each group is expressed in percentage.

A total of 14652 blood donors were sampled for this study. Exchange blood donors were 95%. Majority (14363) of the blood donors were male while 16 donors were females. The donors mean age was 28.6± 2 years. None of the female blood donors had any blood borne infection. Sera from about 229 (1.56%) donors were found reactive for anti-HCV antibodies by Abbott Architect CLIA. Active viremia was confirmed in 138 (0.94%) donors by the gold standard NAT. About 77.19% of reactive donors were in the age range of 19-30 years (Fig.1).

### Co-infections in HCV infected donors

Based on the data of initial screening HBV, HIV, syphilis, malaria and filariasis alone were detected in 288, 89, two, two, and one donor respectively. The HBV infection was found to be the most dominant infection among the donors with a frequency of about 1.96%. Overall, 27.84% of HCV positive donors exhibited co-infection either with HBV (2.89%) or syphilis (26.09%). Triple infection, HIV, malaria or filariasis was not observed in any HCV infected donor.

### Determination of comparative efficacy of MHAA

The MHAA detected anti-HCV antibodies in 199 donors out of these 133 had active HCV infection as confirmed by NAT. The specificity and sensitivity of MHAA is also comparable to CDC recommended serological assays i.e. Anti-HCV Bio-EIA, Monolisa™ HCV Plus V2 and MPD HCV Blot 3.0 respectively (Table-I). The MHAA had an accuracy of 85.57% (95% CI: 82.7-88.06) which was less than the accuracy of Abbott CLIA i.e. 87.90% (95% CI: 85.62-90.22).

**Table-I:** Performance analysis of different tests with PCR as gold standard.

Test	Positive Predictive Value (*95% CI)	Negative Predictive Value (*95% CI)	Sensitivity (*95% CI)	Specificity (95% CI)	Accuracy (95% CI)	**Youden’s J index
Anti-HCV Bio-EIA	72.93% (69.79-76.07)	92.54% (89.91-95.17)	95.09% (92.93-97.25)	63.27% (58.22-67.82)	79.5% (76.64-82.35)	0.58
Monolisa HCV Plus V2	71.22% (68.02- 74.42)	96.72% (95.46- 97.98)	98.01% (97.02-98.99)	59.60% (56.20-62.99)	79% (76.12-81.88)	0.576
Abbot CLIA	71.49% (68.30-74.68)	100%	100%	62.27% (58.83-65.70)	87.90% (85.62-90.22)	0.62
MPD HCV Blot 3.0	79.67% (76.83-82.52)	87.01% (84.64-89.39)	90.74% (88.64-92.78)	72.83% (70.68-74.86)	82.50% (79.81-85.19)	0.64
***MHAA	69.64% (66.39-72.79)	95.19% (93.67-96.70)	96.89% (95.66-98.12)	59.28% (55.81-62.75)	85.57% (82.7-88.06)	0.56

* 95% CI: 95% confidence interval;

** Youden’s J index: Measure of diagnostic accuracy;

*** MHAA: Multisure HCV antibody assay.

## DISCUSSION

Transfusion transmissible infections (TTI) have always been a risk factor in transfusion dependent therapies. Due to lack of compliance with WHO recommended screening strategies and/or lack of proper screening facilities in public healthcare facilities in Pakistan; the TTI still contribute significantly to disease dependent socioeconomic burden for the country. The overall sero-prevalence for HCV infection alone in Pakistani healthy adults is 6.8%.^3^ According to national statistical survey the mean prevalence of HCV among donors at national level was found to be 2.45% while it is documented as 2.31% from the city of Karachi alone.^2^,^15^,^16^ A comparatively lower rate i.e. 0.94% of active HCV viremia in blood donors was observed during the present study. The most common infection among the donors was found to be HBV followed by HCV, syphilis and malaria. It was interesting to note that 36 out of 89 syphilis positive blood donors were presented with HCV coinfection. The frequency of HCV and syphilis coinfection was higher (0.245%) than 0.09% reported by Sial et al., from Lahore.^17^ The demand for infusion of blood and blood products is generally high at medical facilities like NIBD dealing with hematological disorders such as aplastic anemia, leukemias, thalassaemia etc. Owing to such a high demand we are still unable to identify professional donors at NIBD. These professional donors may be a potential carrier of sexually transmitted infection therefore strict screening for TTI is followed to avoid additional post- transplant or post-transfusion complications due to these TTIs.

Post transfusion viral hepatitis due to HCV is a commonly reported viral infection all over the globe. As a preventive measure for transfusion transmitted HCV infection; WHO has formulated a proper guideline for screening of blood and blood products.^12^ The HCV screening protocol includes initial evaluation for anti-HCV antibodies by rapid diagnostic tests in limited care health settings followed by confirmation by CLIA or EIA for HCV core antigen. Samples found reactive by CLIA/EIA need further confirmed by NAT. The NAT is the gold standard for HCV diagnosis with a detection limit of 2-9.4 IU/ml of HCV RNA.^18^ During the present study, among the serological tests, the Abbott CLIA for HCV core antigen was found to be the best. The sensitivity for Abbott CLIA was 100% which is in line with previous studies whereas the specificity was 62.27% (95% CI: 58.83-65.70; Table-I).^13^,^19^ The reason for this lower specificity compared to the reported range of 96-100% might be the high rate of false positive observed during this study.

The MHAA is a third generation serological assay with multiple recombinant HCV antigens from the core, NS3, NS4 and NS5 regions and thus can be useful in overall improved detection of patients exposed to HCV even during the window period.^20^ It is an inexpensive simple qualitative visual rapid test and does not require sophisticated instruments, expensive reagents or time consuming protocols as compared to other tests such as ELISA and NAT. It has a sensitivity of 96.89% ((5% CI: 95.66-98.12) which makes it comparable to Anti-HCV Bio-EIA, Monolisa™ HCV Plus V2 and MPD HCV Blot 3.0 respectively (Table-I). Hence, it can be utilized in resource limited settings with financial constraints; for initial HCV screening. Conversely, there are reports on limitations of this rapid device. Kosack and Nick suggest that very weak lines sometimes cause false result interpretation and needs observer’s expertise to avoid any discrepancy.^21^ Moreover, Poiteau et al. suggest that MHAA cannot perform well with frozen samples since the results for all the frozen samples tested during their study were either negative or indeterminate.^22^ Thus, special care is required when using this device in case of stored samples.

## CONCLUSION

In conclusion, we hereby suggest that both Abbott CLIA and MHAA performed very well in detection of HCV infection in blood donors. However, Abbott CLIA showed a clinical sensitivity of 100% approaching that of HCV RNA detection by NAT, whereas the multi-parametric assay MHAA was significantly less sensitive. The HCV antigen CLIA did not miss any sample positive by NAT whereas MHAA failed to detect a small proportion of samples (5 samples) with active viremia. The WHO recommends use of rapid testing devices in resource constrained countries for initial HCV screening thus MHAA can be effectively used as a cost effective rapid test in such setups for early detection and prevention of further spread of HCV through infected donors.

### Authors’ Contribution

**AN:** Designed project and critically reviewed the final manuscript.

**SNM:** Data acquisition, analysis, literature review, drafted the article, prepared the final version. **IN:** Performed the laboratory tests.

**TSS:** Critically reviewed the manuscript and approved the final version.
